# A Device to Register Temperature in Boreholes in Northwest Spain for Geothermal Research

**DOI:** 10.3390/s22134945

**Published:** 2022-06-30

**Authors:** Germán Marcos-Robredo, Miguel Ángel Rey-Ronco, María Pilar Castro-García, Teresa Alonso-Sánchez

**Affiliations:** 1 Departamento de Energía, Universidad de Oviedo, 33003 Oviedo, Spain; marcosgerman@uniovi.es (G.M.-R.); rey@uniovi.es (M.Á.R.-R.); 2 Departamento de Explotación y Prospección de Minas; Cátedra Hunosa, Universidad de Oviedo, 33003 Ovied, Spain; tjalonso@uniovi.es

**Keywords:** device, error, measurement, temperature, sensor, geothermal, borehole

## Abstract

This paper presents a device used to measure and register temperature for long-term subsoil measurements in boreholes. The borehole of this study is located in Gijón (Asturias, Spain). The measurements were made through two fixed sets of sensors coupled to the geothermal pipe, constituting two independent installations: (a) a commercial device called “Hobo”, which uses TMCx-HD-specific sensors based on resistors with variable resistance; and (b) a device built by this research group, which uses DS12B20 Maxim sensors, a bus 1-wire, and a recording device based on a conventional Arduino board. Temperature was registered every 5 min across several years. These measurements were used to thermally characterize the subsoil, determining the apparent thermal diffusivity, and to study the thermo-hydrogeology of the Lower Jurassic Gijón’s formation made of Liassic limestones and dolomites. This work is part of the Q-Thermie group’s research called “Shallow Thermal Energy”.

## 1. Introduction

Temperature measurement in boreholes is used across a large number of engineering studies [1], such as: (1) the determination of lithologies [2]; (2) the exploration of gas and fluid production engineering [3,4,5]; (3) the exploration of hydrocarbons [6,7] and ore minerals [8,9]; (4) geological and ore prospecting [10]; (5) hydrogeology [11,12,13]; (6) the determination of soil physical parameters [14,15,16]; (7) climate studies [17,18]; and (8) construction and tunnelling [19,20]. However, the most important application is in the geothermal field, which can be found in many papers. The references can be classified based on their purpose:

- To carry out a thermal response test (TRT) or a geothermal response test (GRT) [20,21,22,23].
- To design a thermal energy storage equipment and ground heat exchangers. Ref. [24] used long-term ground temperature records from 20 sample sites scattered throughout five states of Australia.
- Refs. [25,26,27,28] carried out a study of ground temperature distribution between the summer of 1999 and the spring of 2001. Years later, the undisturbed ground temperature over 10 years was recorded [29]. Interesting conclusions about the evolution of subsoil temperature were obtained.
- Other researchers have shown interest in measuring the temperature at different subsoil depths for long periods of time to determine thermal properties [30,31,32,33,34].

The aim of this paper is to show an instrumentation for subsoil temperature measurements during long periods of time, in the order or years, with redundant systems and multiple sensors arranged in the bus 1-wire. The instrumentation features used are detailed, as well as the results of their implementation in the geothermal borehole placed at the Campus of Viesques (Universidad de Oviedo, Gijón, Asturias, Spain).

A geothermal borehole applied to geothermal research is a heat exchange system between the ground and a heat transfer flux that flows inside geothermal pipes installed in the borehole. Moreover, it can provide information about the ground temperature if thermal instrumentation is employed, which is a key aspect in shallow geothermal studies. This last application is the motivation of the current study.

The diameter of a geothermal borehole was bigger than 110 mm, while the diameter of the pipes varied between 32 and 40 mm. The space between the pipes and the borehole was filled with a geothermal grout. These characteristics constrain the instrumentation size and the working conditions.

In the present study, the space was limited so the sensors could be placed in a maximum of 15 mm diameter. This instrumentation can be installed in a permanent setting; in this study, it could circulate through the circuit of the geothermal pipes. In addition, it is interesting to obtain temperature measurements at different depths over long periods of time.

The temperatures measured in shallow geothermal studies can vary between −10 y 50 °C. Nevertheless, temperature fluctuations in the ground depend on depth, its thermal characteristics, and air temperature must be taken into account.

With this in mind, Ref. [30] showed the following, considering that the thermal diffusivity of the ground is 1.2 × 10^−6^ m^2^/s and the amplitude of the seasonal variation of air temperature is 3.8 °C:

- At 5 m deep, the amplitude of the temperature wave is 1.66 °C;
- At 10 m, the amplitude drops to 0.72 °C.

This aspect limits the precision of temperature sensors. In the present study, a 0.1 °C detection limit was established.

The novelty of the designed system is the most remarkable feature, as well as its simplicity. The precision became sufficient for the needs of shallow geothermal studies. The designed system has proven to be highly reliable and robust in a potential aggressive environment, due to water presence, with regards to the pressure at which the system is submitted, etc.

Even though the components and integration described in the current study could be considered somewhat standard, the setup may have the potential to provide future studies with useful insight into building inexpensive and customized data acquisition systems.

## 2. Background

Previous studies have shown that subsoil temperature measurement is of high interest in different scientific fields. In this section, the different systems used to measure and register temperature in geothermal science are analyzed, from the classic (wireline logs) to the more sophisticated ones.

- Systems with probes that move inside a borehole suspended by a cable (wired probe)

Ref. [34] conducted a study with a data logger (pressure and temperature) called RBRduet and presented an inverse numerical model to extend the thermal response test (TRT) assessments to a nearby borehole. The submersible data logger had a fast temperature response; the thermistor accuracy, resolution, and time constant were $\pm 2\times 10^{-3}\;{}^{\circ}C$, $\;5\times 10^{-5}\;{}^{\circ}C$, and $1\times 10^{-1}\;s$, respectively. The pressure sensor could reach a depth of 500 m, and its accuracy and resolution for depth measurements were $2.5\times 10^{-1}\;m$ and $5\times 10^{-3}\;m$, respectively.

Refs. [35,36,37,38,39] introduced an instrument with a data logger to automatically obtain the temperature measurements along the borehole heat exchanger (BHE). This innovative device called Geowire was implemented with a spatial minimum resolution of 0.5 mm, a temperature maximum resolution of 0.06 °C, and an acquisition time of 750 ms. The temperature was monitored with a high-accuracy thermometer of ±0.03 °C accuracy and ±0.01 °C resolution.

- Systems with probes that move inside a borehole without cable (non-wired probes)

A non-wired immersible measuring object for temperature (NIMO T) is described by [40,41,42]. This device consisted of pressure and temperature sensors and a mini-data logger microprocessor used in a closed metal tube, with water-tight pressure reaching up to 100 bars. This instrument had a temperature resolution of ±0.003 °C, and the measurement ran for a depth of 300 m, taking less than 60 min. Recently, it has been used in other studies [43].

Refs. [44,45] demonstrated miniaturized wireless sensors (spherical probe), which used a thermal fluid inside the pipes of the borehole. The characteristics of the autonomous sensor were as follows: temperature range of 0–40 °C; resolution of <0.05°C; accuracy of ±0.05 °C; sampling interval of 0.1–25 s, and sampling capacity of 1000 samples.

- Systems with sensors permanently located in the borehole

The use of optical fiber thermometers (FOs) in geothermal wells was first tested in the late 1980s [46,47]. These instruments measure the temperature distribution along an optical fiber sensor, using the dependence of Raman scattering light’s strength on temperature. These devices have been used in different experiments [48,49,50].

- Systems with sensors combined with other elements of the borehole

Ref. [51] used heating cables inserted into a single pipe of the ground heat exchanger (GHE) to inject heat in the subsurface, based on earlier works [52,53]. In 2018, two thermal response tests using heating cable sections and a continuous heating cable were performed in two experimental heat exchangers in different sites [54].

The cone penetration test (CPT) technique applies a small penetration rod and conducts a line heat source in order to obtain thermal conductivity [55,56,57].

Accordingly, the present study focuses on a simple and novel system designed to measure temperature with sufficient precision to fulfil the needs of the shallow geothermal research.

The designed system is characterized by the fact that it uses temperature sensors placed at different fixed and predetermined depths. These sensors were selected in light of their size, precision, robustness, and installation simplicity, and because they can register and send signals during long periods of time and in the different operational settings of the geothermal borehole.

Two types of sensors were used:

○ Commercial sensors were installed over geothermal pipes, and were embedded in the geothermal grout and connected to the data logger with a wire;
○ The DS18B20 sensors were connected with a one-wire cable which supplies energy to the sensor and transmits a signal to a specifically designed data logger, placed outside the borehole. This is a novel and simple setting, which has proven to be useful as temperature data are registered for a long period of time.

## 3. Materials and Methods

Firstly, suitable temperature sensors were sought, considering that they had to meet the above-mentioned requisites. Hence, the precision, repeatability, and calibration, both in the lab and in the field, were analyzed for ranges of temperature expected in a shallow geothermal borehole.

The connection type of the sensors and of the measurement’s storage was chosen and designed.

The sensors were calibrated in the lab and the different setting types were laid out. Once the sensors were set up in two research boreholes at different positions with respect to the geothermal pipe, measurements were taken. The aim was to analyze the thermal jumps with the two types of sensors and the effects of their position inside the borehole on the temperature measurements.

**Figure 1:**
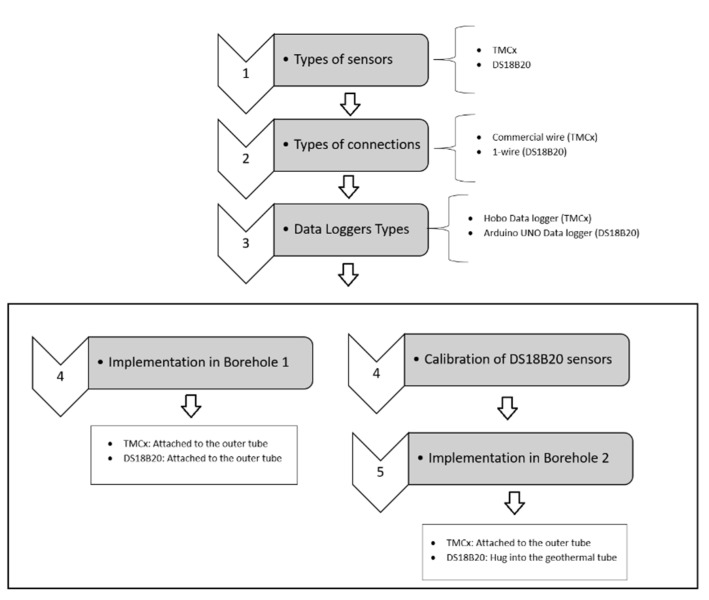
Flow chart.

Figure 1 shows a flow chart of the steps taken.

A detailed description of the technical aspects is provided, starting with the characteristics of the boreholes where the sensors were placed. Then, graphs showing the temperature evolution over a year of measurements are presented. These graphs emphasize the validity of the designed instrumentation and consider the base of shallow geothermal studies.

### 3.1. Boreholes and Geothermal Instrumentation

Below, the instrumentation used in the Q-Thermie-uniovi research boreholes 1 and 2 for the current study is detailed.

#### 3.1.1. Boreholes Q-Thermie-Uniovi 1 and 2

**Figure 2:**
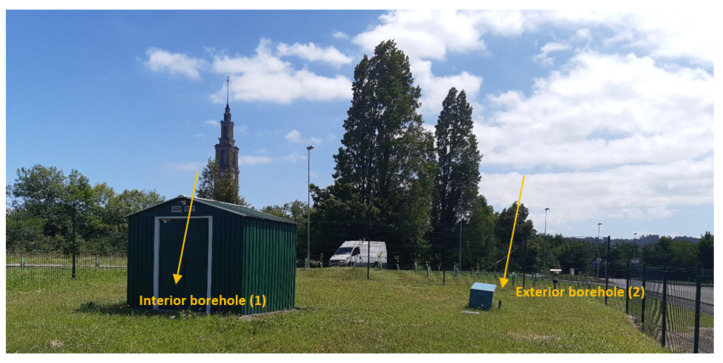
Location of the Q-Thermie-uniovi research boreholes at the Campus of Viesques (Gijón, Asturias).

The Campus of Viesques was formed by several lecture rooms, departments headsquares, laboratories, and other services, as well as parks and green areas. The geothermal research boreholes were drilled in one of these green areas (eastern part of the campus), next to a weather station (property of the State Meteorology Agency—AEMET) (Figure 2).

##### Geothermal Borehole “Q-Thermie-Uniovi-1”

In 2012, a geothermal borehole was drilled and a shed was installed over it. Inside this shed, the equipment needed for the research was located. The borehole had a diameter of 125 mm (*Db*) and a depth of 48 m (h), and it was drilled with a down-the-hole hammer using a roto-percussion method.

Coaxial geothermal pipes were chosen due to the small diameter of the borehole. These pipes consisted of an outer tube with a diameter of 50 mm (*D*0) and a concentric inner tube with a diameter of 25 mm (*Di*). The length of exterior pipe was 48 m, and it was closed at the bottom. The inner concentric pipe was open at the bottom to allow the fluid that descends through the central part to rise again to the surface through the annular part.

**Figure 3:**
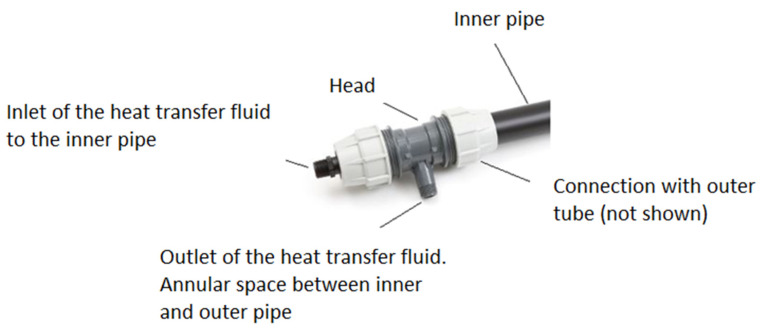
Inner tube of the coaxial pipe and connection head to the outer pipe and the heat transfer fluid circuit.

On the outside of the borehole, both pipers were connected by a head, as shown in Figure 3.

The reasons why the geothermal probe was chosen instead of the U-shape pipe are as follows:

- Easy to assemble: a coaxial geothermal pipe is easier to insert compared to the U-shape pipe. On the one hand, for U-shape pipes, several pipes must be lowered simultaneously, which are joined with an “elbow” at the bottom of the borehole, where a counterweight is also located to facilitate the descent. The coaxial pipes are descended using the following steps: firstly, a unique pipe is inserted—in this case, the outer tube (close at the bottom); secondly, the inner pipe is easily introduced as the outer tube protects it. On the other hand, the use of a head as a linking element between the geothermal pipes and the outside installation of the borehole simplifies the connections outside the borehole.
- Usage flexibility: various configurations can be used—for example, the inner pipe can be removed and changed, or new instrumentation can be inserted.
- Space availability and accessibility for the instrumentation: the installation of temperature sensors in the space between the outer pipe and the borehole walls can be measured, which descends easily at the same time as the outer pipe.

The annulus between the borehole and the outer tube of the coaxial pipe was filled using a dense pre-measured grout with high thermal conductivity and a suitable rheology for injection. This grout is designed especially for geothermal applications, and it was provided by Energrout Geothermal S.L.

This grout is composed of a mixture of silica and with an ideal grading curve, and a sulfur-resistant cement that is enhanced with natural and synthetic additives [30].

The geothermal instrumentation attached to the outer tube, and embedded in the geothermal backfill, was located inside a tube with an inner diameter (*ID*) of 8 mm and an outer diameter (*OD*) 10 mm, as explained later.

**Figure 4:**
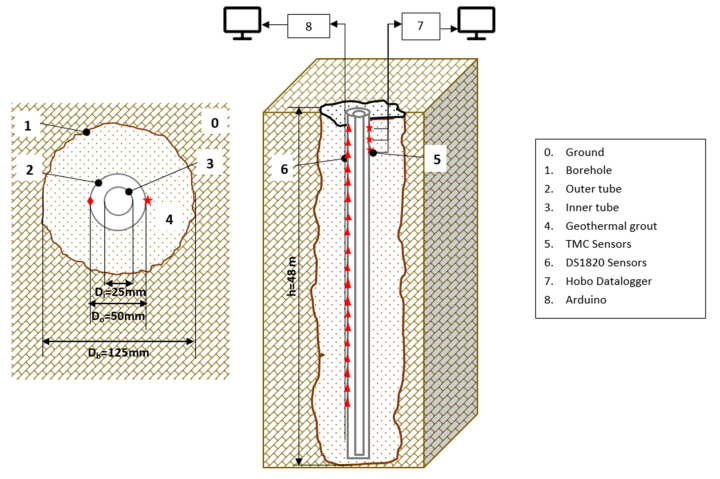
Experimental set-up showing the geothermal borehole, coaxial pipe, temperature sensors, grout, and a cross section of geothermal borehole Q-Thermie Uniovi-1 (figure not to scale). Red triangle corresponds to number 6. Red star corresponds to number 5.

The scheme of the borehole is shown in Figure 4.

This borehole was set up to monitor the internal temperature, as follows:

- Commercial TMCX-HD temperature sensors placed on the external wall of the outer geothermal tube;
- Commercial DS18B20 temperature sensors placed on the external wall of the outer geothermal tube.

Later, the technical specifications of the instrumentation used are described in detail.

##### Geothermal Borehole “Q-Thermie-Uniovi-2”

**Figure 5:**
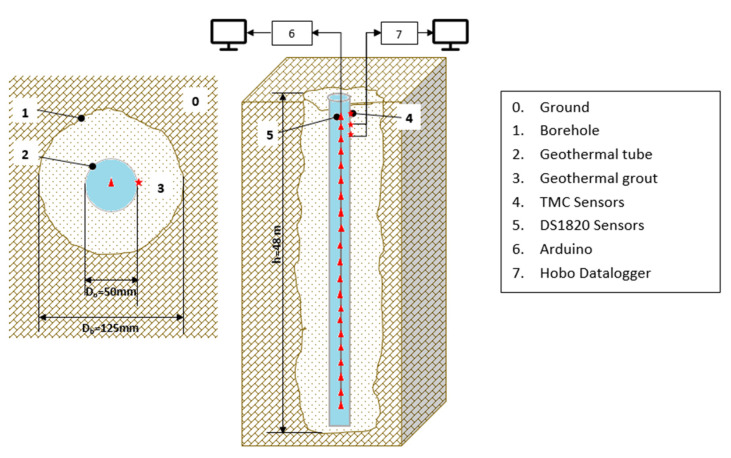
Experimental set-up showing the geothermal borehole, pipe, temperature sensors, and a cross section of geothermal borehole Q-Thermie Uniovi 2, (figure not to scale). Red triangle corresponds to number 5. Red star corresponds to number 4.

In 2018, instrumentations were installed in a borehole previously drilled, located outside at 7.3 m of the Q-Thermie-Uniovi 1 borehole. This borehole was drilled using a rotation method and had a diameter (*Db*) of 125 mm and a depth of 48 m (h). This borehole had an outer geothermal tube with a diameter (*D*0) of 50 mm, and the inside was filled with water. The scheme of this borehole is shown in Figure 5.

Like for the previous borehole, two types of sensors were set up to measure the temperature evolution during long periods of time:

- A commercial TMCX-HD temperature sensor placed on the exterior the geothermal tube;
- A commercial DS18B20 temperature sensor hung into the geothermal tube from bus 1-wire.

Technical specifications of the instrumentation used are described below.

### 3.2. Temperature Sensors and Its Connection

Two types of temperature sensors were used in the current study, as described below.

#### 3.2.1. TMCx-HD Sensors

The TMCX-HD sensors are used in the most superficial part of the boreholes and can be used in water, soil, and air. The features of TMC sensors, according to the manufacturer [58], are as follows:

- Dimensions:$\;5.1\times 33{\;\mathrm{mm}}^{2}$;
- Weight: 37 g;
- Measurement range: −40 °C to +50 °C in water/soil;
- Measurement range: −40 °C to +100 °C in air;
- Response time in air: 2 min typical to 90%, in air moving 1 m/s;
- Accuracy with HOBO U-12: ±0.25 °C. from 0 °C to 50 °C;
- Resolution with HOBO U-12: 0.03 °C at +20 °C;
- Housing: copper-plated sensor tip.

**Figure 6:**
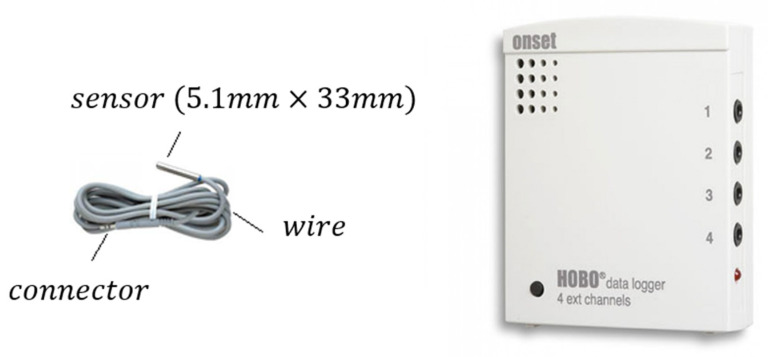
TMCx-HD sensor and HOBO data logger [58,59].

Figure 6 shows the sensor, the connection cable, and the connector. The cable allowed the sensor to be connected with the data logger HOBO (model U-12-006), as described in Section 3.3.1.

##### Connection of the TMCx-HD Sensors to the Data Logger

TMCx-HD sensors were fixed to the outer tube and each sensor cable attached to the tube ascended up to the surface, where they were directly connected to the HOBO data logger.

They were placed at three different depths (Table 1), up to the maximum depth allowed by the sensor.

#### 3.2.2. DS18B20 Sensors

The DS18B20 digital thermometer (manufactured by Maxim Integrated) supplied 12-bit temperature readings. The information was sent to/from the DS18B20 over a 1-wire interface, so that only one wire needed to be connected from a central microprocessor to the DS18B20.

The power required for reading, writing, and performing temperature conversions can be derived from the data line itself without needing an external power source.

Since each DS18B20 sensor had a unique silicon serial number, multiple DS18B20s could exist on the same 1-wire bus. Thus, temperature sensors could be placed in different places.

According to the manufacturer, DS18B20 features are as follows:

- The unique 1-wire^®^ interface requires only one port pin for communication;
- Measures temperatures from −55 °C to +125 °C (−67 °F to +257 °F);
- Measures ± 0.5 °C accuracy from −10 °C to +85 °C;
- Measures programmable resolution from 9 bits to 12 bits;
- No external components are required;
- Parasitic power mode requires only 2 pins for operation (DQ and GND);
- Simplifies distributed temperature-sensing applications with multidrop capability;
- Each device has a unique 64-bit serial code stored in on-board ROM.

**Figure 7:**
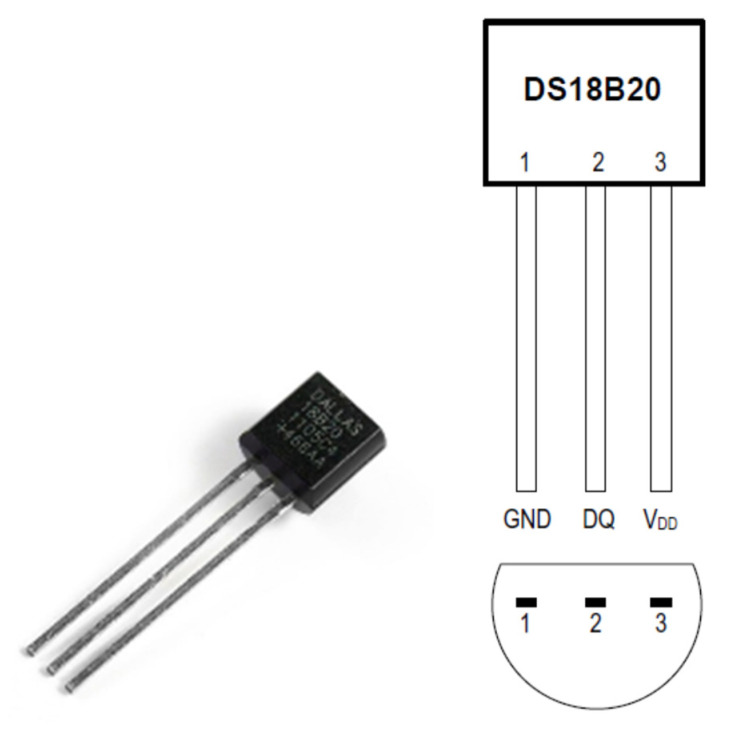
DS18B20 features [60].

Figure 7 shows a DS18B20 sensor and its block diagram. Evidently, DS18B20 sensors, encapsulated with TO-92, have three pins: the power supply VDD (5 V), the reference GND (0 V), and the pin for data collection DQ.

As mentioned before, the DS18B20 sensor can work without external power supply. The power was supplied through the DQ terminal when it had a high value, instead of being provided through a pull-up 1-wire resistance. The high bus signal also supplied an internal capacitor (CPP), which in turn supplied the “device” when the “bus” was low. This method of deriving energy from a bus 1-wire is known as parasite power. This connection means that a 1-wire can be created with only two cables—one for the data (DQ) and power supply and another for the ground GND, without needing a third cable for the power supply.

**Figure 8:**
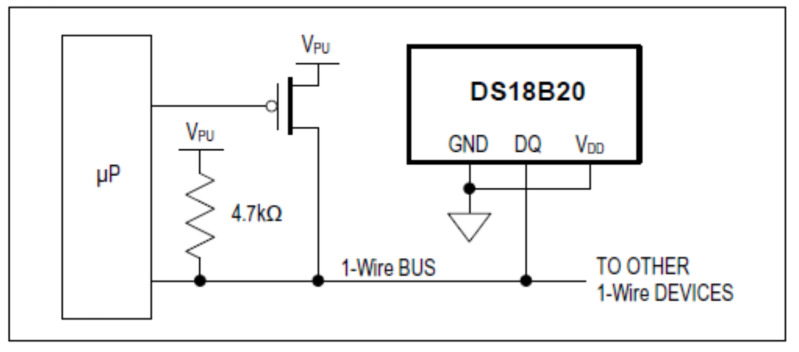
Parasite power connection graphic representation [60].

The parasite power connection is shown in Figure 8.

**Figure 9:**
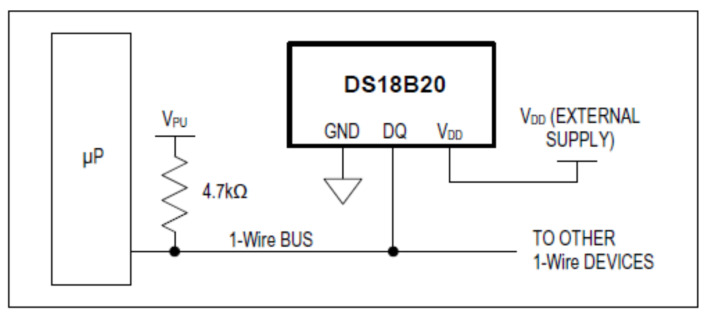
External power supply graphic representation [60].

DS18S20 sensors can also be powered by a VDD external source as an alternative. In that case, the VDD must be connected to the positive voltage, between 3.0 and 5.5 V. The GND ground pin must be connected to the ground (0 V) and the data collection DQ (input/output) must be connected to a pull-up weak resistance of 4.7 kΩ and to the microcontroller pin, which is responsible for reading the temperature measured by the sensor. In this case, the temperature recording was made with specific software for the ARDUINO UNO. Figure 9 shows a graphic representation of the external power supply source.

##### Bus 1-Wire: DS18B20 Sensors Connection

The DS18B20 sensors were connected with an external power supply, using three cables with only 1-wire.

**Figure 10:**
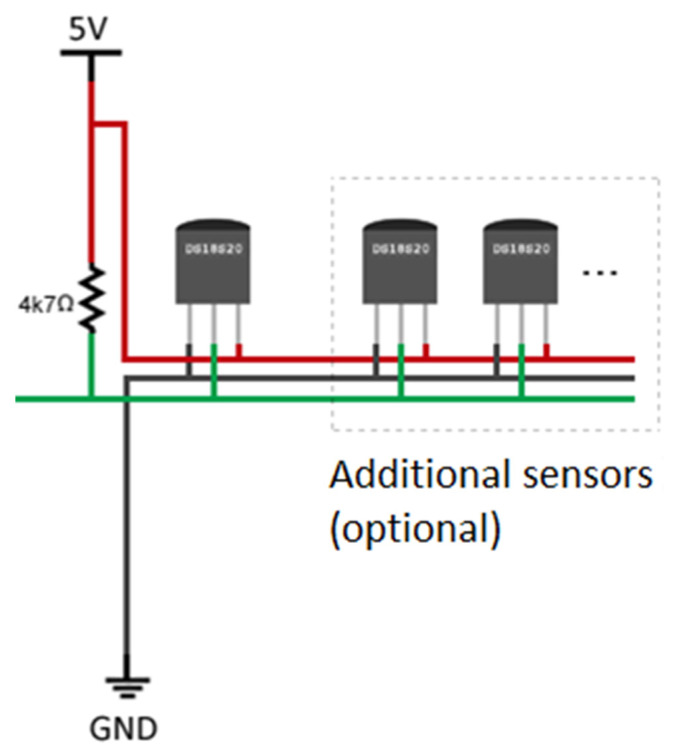
DS18B20 sensor connection diagram to the 1-wire bus.

To minimize the wiring of the DS18S20 sensors, as there were more than 20 sensors in the borehole, which were mounted in a bus of 45 m, with only three lines. The bus used was the 1-wire from Maxim. The three lines were: power, signal, and ground, which were shared with all the sensors connected to the bus. Each sensor pin was soldered to one of the wires, with a connection scheme similar to the one shown in Figure 10. The weld was protected with lined heat shrink tubing.

**Figure 11:**
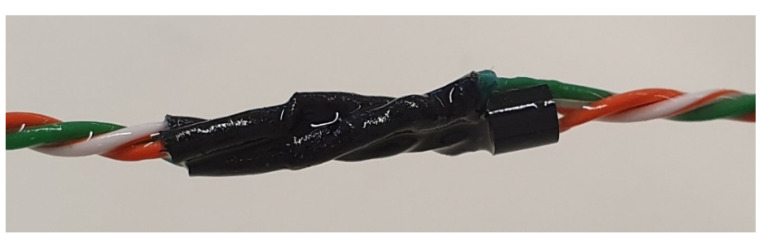
Sensor located on the 1-wire cable.

Figure 11 shows the sensor protected and joined to the 1-wire.

**Figure 12:**
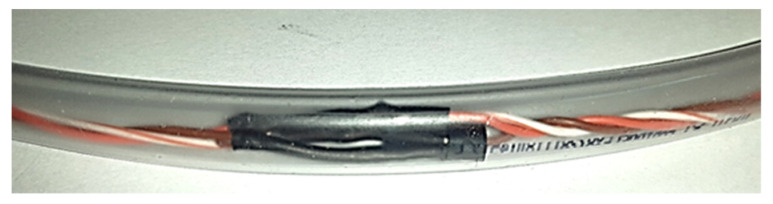
DS18B20 sensor on the 1-wire bus inside the protection hose.

The wire-1 and a set of sensors were introduced in a PVC monolayer hose, which can tolerate high pressures and demonstrate high flexibility at low temperatures. This assembly protects the sensors against pressure and water effects. This hose had an external diameter (*ED*) of 10 mm and an inner diameter (*ID*) of 8 mm (Figure 12).

The hose was filled with vegetable oil, due to its biodegradable characteristics, and then it was sealed at the part that will be located at the bottom of the borehole.

Similar 1-wire devices were manufactured for each borehole, although with different sensor separations. In the Q-Thermie-uniovi-1 borehole, the sensors were located every 5 m, while in the Q-Thermie-Uniovi-2, the sensors were separated 2 m. The distance value slightly fluctuated between each of the sensors in a set, following the manufacturer recommendations of not using equal or multiples distances, potentially leading to communication errors.

The depths at which the sensors were located in both boreholes are shown in Table 2, as well as the distance differences between sensors.

**Figure 13:**
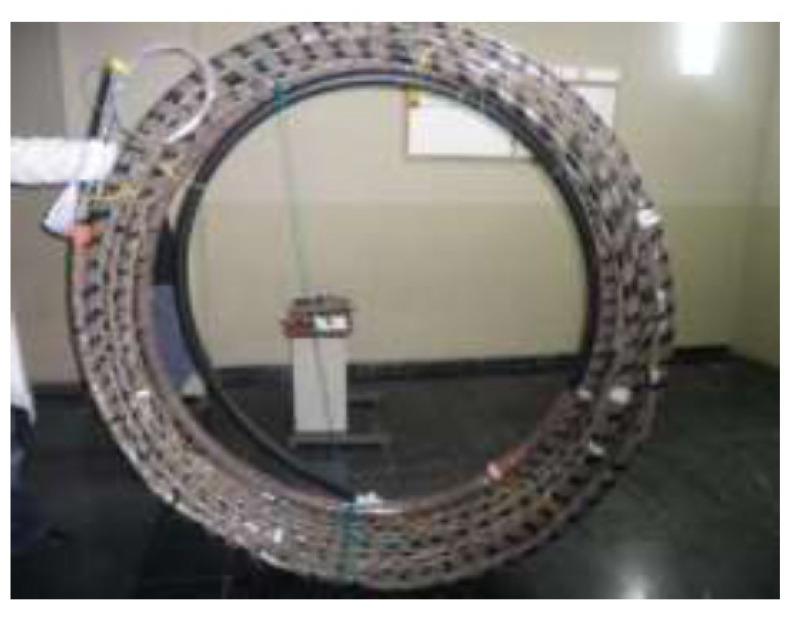
External geothermal pipe with temperature sensors used in Q-Thermie-Uniovi-1 borehole.

In the Q-Thermie-uniovi-1 borehole, a set of sensors was adhered to the exterior part of the tube. Figure 13 shows the exterior tube, which was prepared at the laboratory.

**Figure 14:**
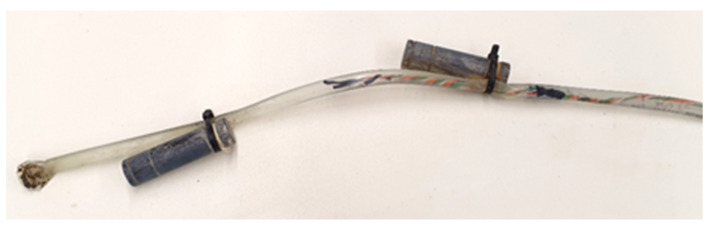
The 1-wire cable protected with an outer tubing and filled with oil Q-Thermie-uniovi-2.

In the Q-Thermie-Uniovi-2 borehole, a set of sensors hung freely and vertically in the axis of the borehole, with weights that ballast the 1-wire (Figure 14).

Outside of the borehole, the 1-wire bus was connected to a microprocessor Arduino UNO, which supplies power to the sensors and registers the measured data.

The cable that joins all the sensor data pins (DQ) was connected to one of the digital entries of the Arduino UNO register and, through the 1-wire bus communication protocol, the data of each sensor were recorded. Similarly, a cable joined all the pins VDDç and another joined all the GNDs. A 4.7 KΩ resistance was located between the cable that joins all the data pins and the one that joins all the power pins. Later, it was seen that communication improved for resistances < 330 Ω.

### 3.3. Data Logger Types

#### 3.3.1. HOBO U12-006 Data Logger

The HOBO data logger is one of the cheapest and reliable systems used to measure temperature, and it is widely used in fieldwork campaigns [61,62].

The HOBO U12-006 data logger collects measurements of up to four TMCx-HD sensors. It registers temperature, relative humidity, and light intensity. With a 12-bit resolution, this data logger could gather a range of recorded data and stores 43,000 measurements.

The HOBO data logger features according to its manufacturer are:

- Analog channels: 0 to 2.5 Vdc (w/CABLE-2.5-STEREO), 0 to 5 Vdc (w/CABLE-ADAP5), 0 to 10 Vdc (w/CABLE-ADAP10), and 4–20 mA (w/CABLE-4–20 MA);
- Accuracy (logger only): ±2 mV ± 2.5% of absolute reading and ±2 mV ± 1% of reading for logger-powered sensors;
- Resolution: 0.6 mV;
- Sample rate: 1 s to 18 h, user-selectable;
- Time accuracy: ±1 min per month at 25 °C (77 °F);
- Operating range: −20 to 70 °C (−4 to 158 °F);
- Operating temperature: −20 to 70 °C (−4 to 158 °F) (logging), 0 to 95% (RH (non-condensing));
- Launch/readout: 0 to 50 °C (32 to 122 °F), per USB specification;
- Humidity range: 0 to 95% (RH (non-condensing));
- Battery life: 1-year typical use;
- Memory: 64 Kbytes (43,000 12-bit measurements);
- Weight: 46 g (1.6 oz);
- Dimensions: 58 × 74 × 22 ${\mathrm{mm}}^{3}$ (2.3 × 2.9 × 0.9 ${\mathrm{inches}}^{3}$).

Figure 6 shows a HOBO data logger reproduction.

#### 3.3.2. Arduino UNO Data Logger

The Arduino, used as a microcontroller for instrumentation, is not only cheap but also accurate. For this reason, it has been used in several studies [63,64].

**Figure 15:**
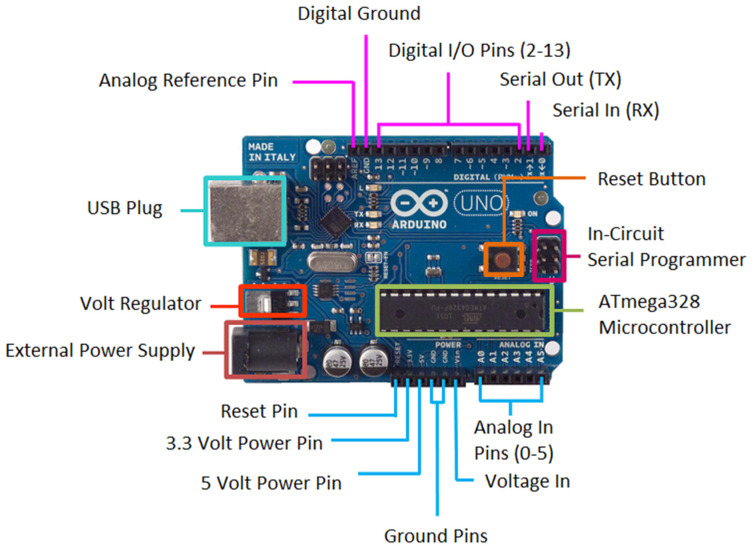
Arduino UNO data logger.

The DS18B20 sensor 1-wire set was connected to an Arduino UNO, which is a free software and hardware platform based on the ATmega microcontroller, used both for product development and education. Figure 15 shows an Arduino UNO data logger.

The Arduino UNO was powered to 7 V by a direct current power supply connected to the 230 V power grid. It was also possible to connect the Arduino UNO to an external battery between 6.5 and 12 V, but due to its high consumption, this application was not feasible.

The digital pin 2 on the Arduino was used as the entry for reading data from the 1-wire. In this communication protocol, developed by Dallas Instruments, one of the sensors acts as a master and the rest as slaves.

For the 1-wire power, the Arduino UNO provided a constant 5 V output, which was connected to the wire that connects the VDD pins of the sensors. This value was within the range recommended by the manufacturer (3–5.5 V), for powering DS18B20 sensors.

The resistance recommended by the manufacturer to reduce communication errors was located between the wires that join the VDD and the DQ pins from the sensors. Due to the cable length and the welds of the sensors to the cables, the 4.7 kΩ pull-up resistance was replaced by a 330 Ω one, reducing communication errors.

A program was developed to register temperatures of 1-wire sensors for every time interval, as predefined in Table 2.

**Figure 16:**
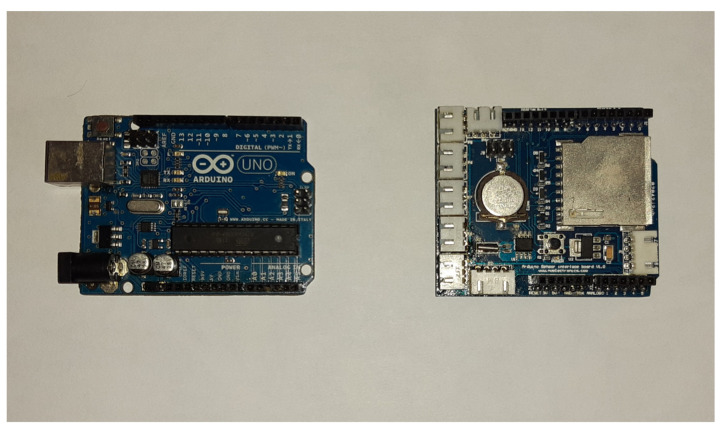
Arduino UNO card and Shiel SD Nuelectronics card (from Nuelectronics.com, accessed on 1 January 2020).

The time control was carried out with a Shield SD card (from Nuelectronics.com, accessed on 1 January 2020) attached to the Arduino, as it had a real time clock (RTC) and a SD card reader. The RTC was required for time and date registration, and the SD card was used for storing large amounts of generated data (Figure 16).

The Shield SD card was powered by the Arduino UNO itself, through the 5 V GNS, but it had a 3 V CR1220 button cell which was used to keep the card’s RTC operational if the 7 V power supply failed.

It should be emphasized that it was possible to read the data collected on the SD card while the system was operational. The SD card was then read in an external device, reinserted, and the system was finally restarted. This was not an issue for borehole monitoring with a sampling period of 5 min, as demonstrated in the present study. Nevertheless, this could be of importance for similar systems with a higher sampling frequency.

**Figure 17:**
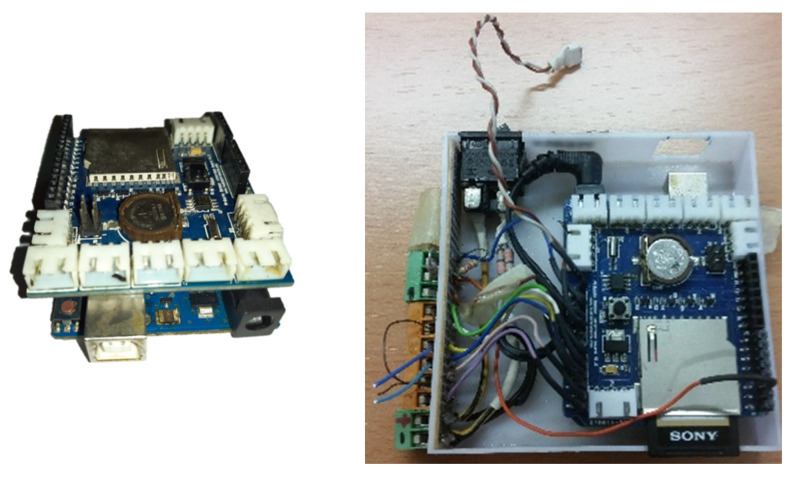
Arduino assembly with Shiel SD card and the final device adapted to field work.

Figure 17 shows the assembly of both cards, and their final arrangement in a box, where the connections were wired to facilitate their placement in the proximity of the borehole.

The final look was very compact and adapted to the working conditions. The assembly measures 92 × 92 × 40 ${\mathrm{mm}}^{3}$.

### 3.4. Individual Calibration of DS18B20 Sensors

#### 3.4.1. General Sensor Calibration

**Figure 18:**
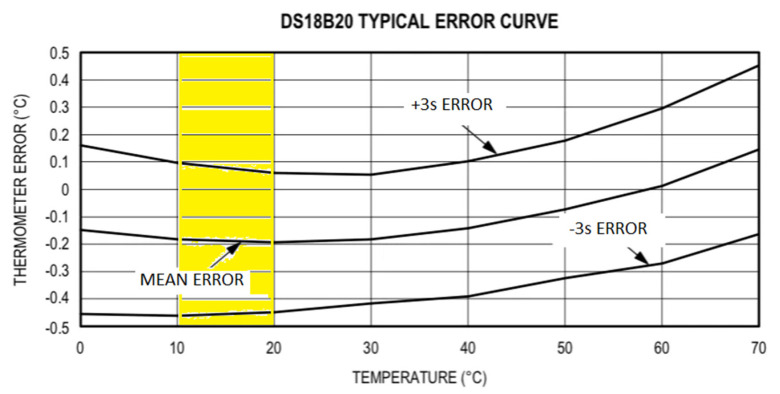
Typical error curve [60]. The temperature zone of interest in the current study is highlighted in the box.

The general sensitivity of the DS18B20 sensors was provided by the manufacturer and is shown in Figure 18.

The range in which the measurements of the borehole sensors oscillate is highlighted. In this case, according to the sensor’s calibration, the mean error was −0.2 °C (for a confidence interval of 99.7%, the values ranged between −0.45 °C and 0.1 °C). The manufacturer also indicates that this sensitivity can be improved through individual calibration for each sensor.

In the current study, individual calibration was carried out, as detailed below, forcing the temperature of sensors to range between 10 and 20 °C.

#### 3.4.2. Individual Sensor Calibration

The individual calibration was carried out for each DS18B20 sensors in the 1-wire located in Borehole 2. As each sensor was located at a depth *z*, they could be identified as $S_{z}$ and the temperature was registered as $T_{z}$.

Twenty-one sensors were individually calibrated once the sensors were installed on the 1-wire bus and the set of cables inside the protective monolayer PVC hose was introduced.

For the calibration, the following operations were executed:

(1) The tube with the 1-wire was introduced into a thermal bath;
(2) The 1-wire was connected to the Arduino UNO, as indicated above, and equipped with the data reading/registering program;
(3) Temperature variations were registered for 5 min and several hours in each sensor. The thermal bath temperature varied, while registering the sensor readings. This defined several calibration zones with different temperatures.

**Figure 19:**
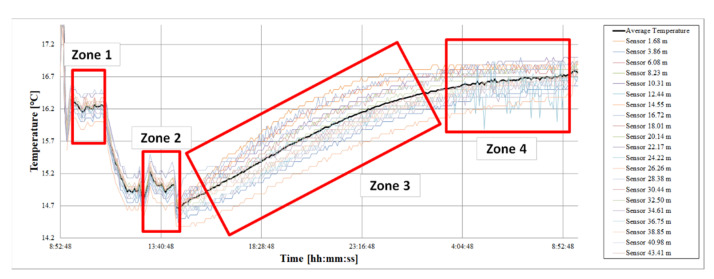
Graphic representation of the four stable zones detected during the sensor calibration process.

Figure 19 represents the evolution of temperature readings during the calibration period for each of the 21 DS18B20 sensors that make up the 1-wire. The mean temperature $\left(T_{m}\right)$ of the set of sensors at each moment was calculated. $T_{m}$ was considered to be the true temperature of the thermal bath.

Table 3 summarizes the $T_{m}$ values registered in each of one of the zones. At the beginning of the study, the bath was kept at a stable $T_{m}$ of 16.24 °C (zone 1); after a certain time, it was lowered to 14.98 °C, allowing the entry of water from the network in the thermal bath. The network water was colder (zone 2). Subsequently, the water entrance was suppressed, so that the temperature of the bath gradually rose (zone 3) until it was stabilized again (zone 4).

**Figure 20:**
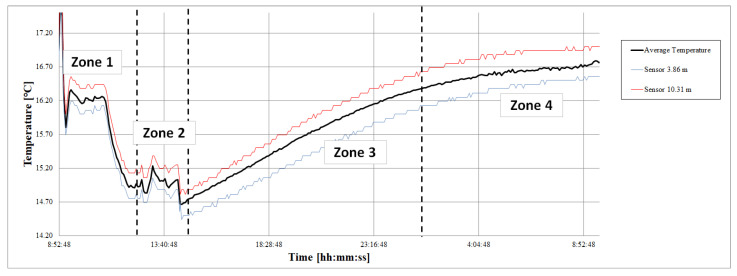
Temperature measured by S3.86 and S10.31 sensors, characterized by maintaining the deviation trend from the mean.

Each of the sensor’s $T_{m}$ at each zone were compared with the mean temperature in that zone. For simplicity, Figure 20 represents the temperatures of two sensors: the mean temperature of all the sensors $\left(T_{m}\right)$ in black, and the temperatures $\left(T_{z}\right)$ for z=3.86 m and z=10.31 m in blue and red, respectively. The sensor located at 3.86 m was always measured below the mean temperature, while the sensor located at 10.31 m measured above the mean temperature in all the zones.

**Figure 21:**
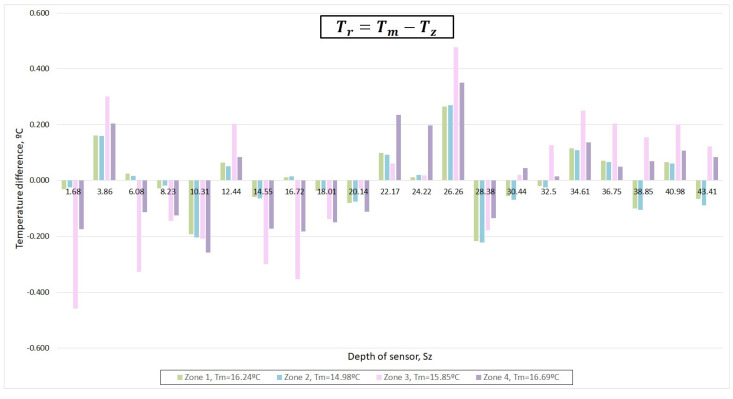
Difference in the mean temperature from each sensor in each zone, according to the mean temperature of the bath.

Figure 21 shows the temperature difference (ΔT) between the mean temperature of the set of sensors ($T_{m}$) in the period corresponding to a zone and the mean temperature recorded by the sensor $(T_{z}$) in the same period. Positive values of ΔT were obtained when the sensor measured by default. At the bar diagram representation, the value of ΔT is shown for each sensor (identified by the depth at which it is located) and each zone (represented by a color of the bar).

As seen in Figure 20, the sensor located at 3.86 m had positive ΔT (it measures by default), while the sensor located at 10.31 always measured in excess (negative ΔT).

The trend in the deviation tended to be maintained. However, in the sensors located at 6.08, 16.72, 30.44, 32.55, 38.85, and 43.41 m, the deviation oscillated between positive and negative (same sensor and different zones). In Figure 21, a temperature variation in these sensors is shown.

**Figure 22:**
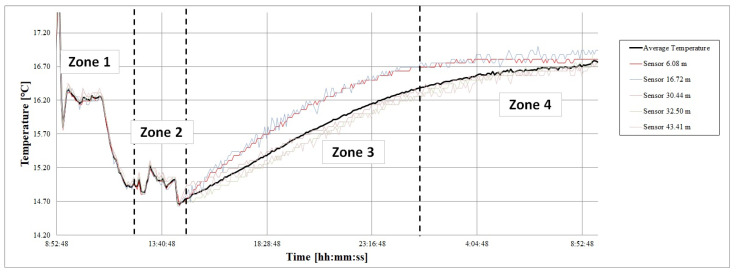
Measured temperature by different sensors characterized by not maintaining the deviation trend from the mean.

**Figure 23:**
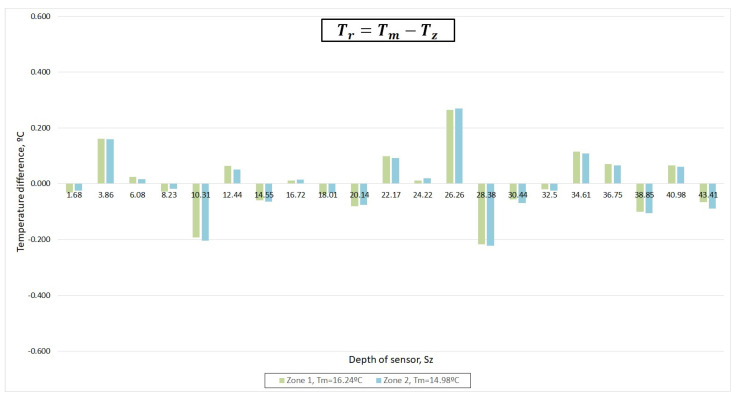
Measured temperature deviation of each one of the 21 sensors of 1-wire with respect to the mean temperature in zones 1 and 2.

Figure 21 and Figure 22 shows that the most important deviations increased in zones 3 and 4, while the sensors behaved in a stable way in zones 1 and 2. Therefore, calibration was performed using zones 1 and 2. Figure 23 also verifies this deduction.

Calibration was carried out with a linear regression between the set of values *T_z_*(*t*) vs. *T_m_*(*t*) in zones 1 and 2.

**Figure 24:**
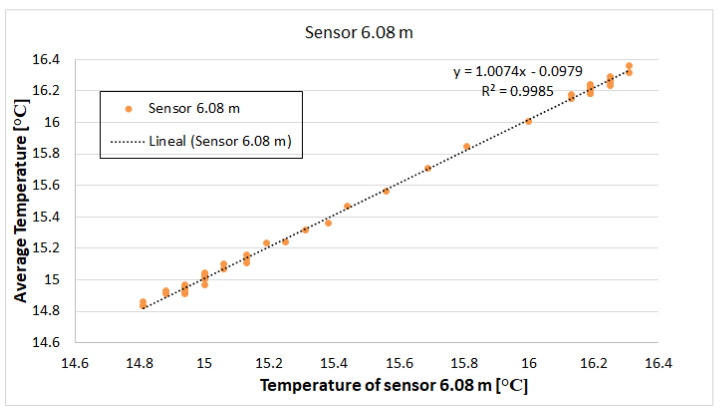
Calibration line for sensor DS18B20 located at 3.86 m.

In Figure 24, the *T_m_* of all the sensors at a given instant was plotted against the temperature of the sensor *T*6.08 at the same instant. The data set was adjusted to the equation: $T_{m}=a*\;T_{6.08}+$ *b*, with *R*^2^ = 0.9985. This demonstrates the calibration line for this sensor. The coefficients of *a* and *b* were 1.007 and −0.0979, respectively.

This calibration procedure was repeated for each of the 21 sensors. Table 4 shows the parameters resulting from this calibration.

With this calibration, the DS18B20 sensors of the “Q-Thermie-uniovi 2” borehole were corrected for their tendency to deviate the measurement towards lower or higher values of the true value.

## 4. Results

Following, the results from the instrumentation applied in the research boreholes described in Section 3 are detailed.

### 4.1. “Q-Thermie-Uniovi-1” Borehole

The results obtained for both types of sensors are presented. The DS18B20 sensors were uncalibrated; hence, the data obtained were those collected by the data loggers. The repeatability of the measurement was analyzed; the temperature differences registered by both types of instrumentation were measured and the potential causes of the differences were analyzed.

#### 4.1.1. TMCx Sensors

**Figure 25:**
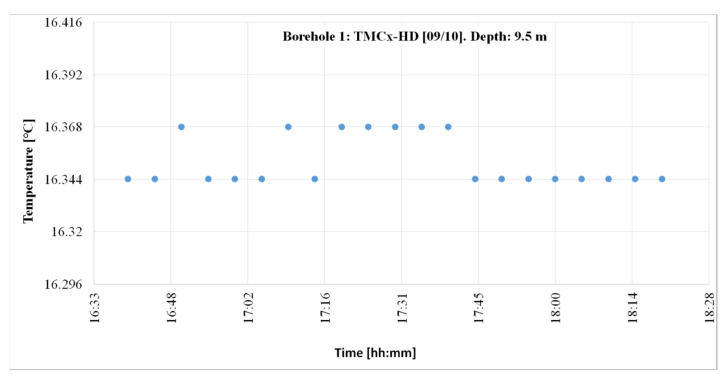
Temperature registered on 9 October 2012 at the “Q-Thermie-Uniovi-1” borehole with TMCx-HD sensors.

Figure 25 shows the temperature registered by the TMCX-HD sensors in 2012 and at 9.5 m (embedded between the geothermal fill and attached to the outside of the outer tube). The values were obtained during a thermal response test (TRT) when the ground temperature was unperturbed. The temperature registers took place from 4:40 to 6:20 p.m., every 5 min.

Observably, 66.6% of the registers were centered on 16.344 °C and the remaining 33.33% were centered on 16.368 °C. The thermal jump was 0.024 °C. The registering period lasted approximately two hours, and measurements were registered every 5 min. The number of data read in the period was 21.

It is worth acknowledging that these sensors continued to work properly.

#### 4.1.2. DS18B20 Sensors

**Figure 26:**
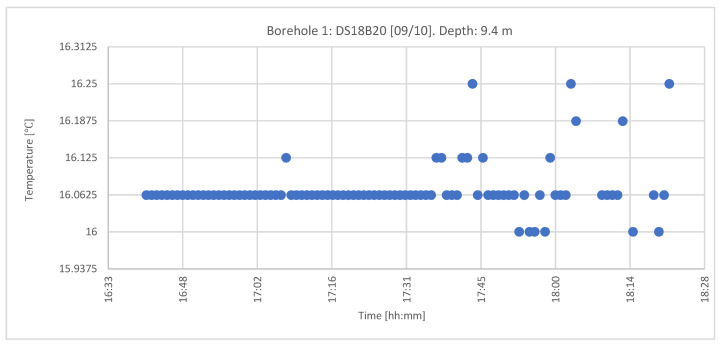
Temperature records from 9 October 2012 at the “Q-Thermie-Uniovi-1” borehole with DS18B20 sensors.

Figure 26 shows the registered and corrected temperatures from the DS18B20 sensors embedded between the geothermal fill (attached to the outer concentric geothermal pipe), similar to the previous sensors. The temperature registers took place on the same date, from 4:41 to 6:22 p.m., and the measurement interval was 1 min, corresponding to a depth of 9.4 m. The number of data was 101.

After 6:00 p.m., the thermal jump fluctuated and increased significantly, until the sensors stopped working. This was caused by the entry of water inside the pipe that housed the sensors.

**Figure 27:**
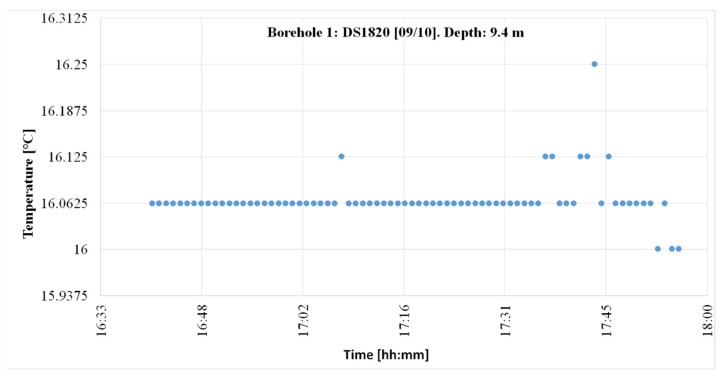
Temperature records from 9 October 2012 at the “Q-Thermie-Uniovi-1” borehole with DS18B20 sensors excluding data.

Figure 27 shows the temperature log after the post-fault logs were ignored.

**Figure 28:**
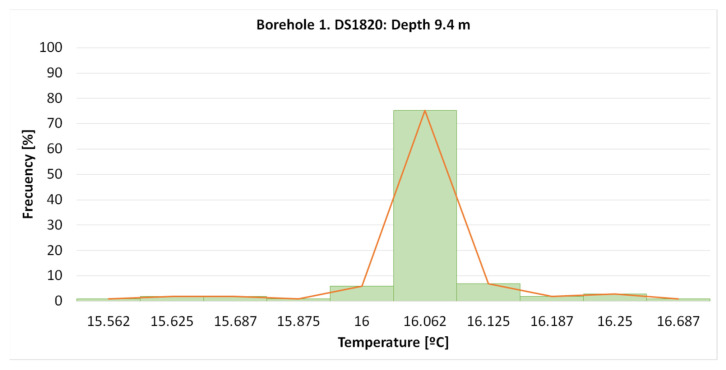
Histogram and frequency graph of DS18B20 sensor located at 9.4 m.

Figure 28 shows that the measurements collected in this period, one each minute, were centered on 16.062 °C, in 75.2% of the cases. Occasionally, there was a thermal jump of 0.062 °C.

#### 4.1.3. Comparative Study between the Measurements of Both Sensors Located at a Similar Depth and with the Same Ubication in the Pipe

Both sensors were located on the outer wall of the geothermal probe and were embedded in the geothermal fill. The depth at which they were found was similar, with a 0.1 m difference, i.e., the TMC sensor placed at the deeper point.

The measurement period was the same, although the number of observations was different because the temperature was registered every minute with the DS18B20 sensors, while it was measured every 5 min in the TMC. Therefore, the observations in the first case were 101, while the number of observations with TMCs was 21.

It is observed that the TMC sensor (9.5 m) measured 0.282 °C higher than the DS18B20 sensor (9.40 m). This could be due to depth differences, different sensor technology, and/or because the DS18B20 sensors were not calibrated.

According to [33], during the period when temperatures were measured (October), the deepest sensor (TMC) was expected to measure a temperature slightly lower than that of the shallowest one. Therefore, it can be concluded that the difference between both sensors was mainly due to a lack of calibration within the DS18B20 sensors.

Table 5 shows the temperature comparison between the two sensor types, placed at a different position and similar depth (in borehole 1)

### 4.2. “Q-Thermie-Uniovi-2” Borehole

The DS18B20 sensors in this borehole were calibrated; therefore, the calibration influence was analyzed, in addition to the other aspects previously mentioned.

#### 4.2.1. TMCx Sensors

**Figure 29:**
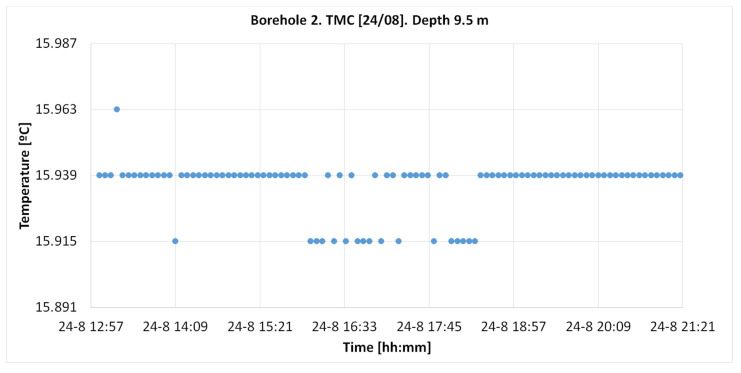
Temperature register on 24 August in the “Q-Thermie-Uniovi.2” borehole with TMCx-HD sensor at 9.5 m.

Figure 29 shows temperature measurements from the TMCx-HD sensors (attached to the exterior of the outer geothermal pipe), from 01:05 to 09:35 pm, taken every 5 min, at a depth of 9.5 m. The total number of considered measures was 103.

In this case, 82.5% of the measurements reached a value of 15.939 and 16.5% reached a value of 0.024 °C, differing from the main value.

#### 4.2.2. DS18B20 Sensors

The 1-wire of this borehole was calibrated; hence, the analyzed temperatures were the corrected ones.

There was no sensor located at the depth of 9.5 m where the TMC sensor was placed. Therefore, the results from the DS18B20 sensors located at depths closest to 9.5 m were analyzed (8.23 and 10.31 m). Finally, the results from both sensor types were compared.

##### Temperatures from Sensor S8.23

**Figure 30:**
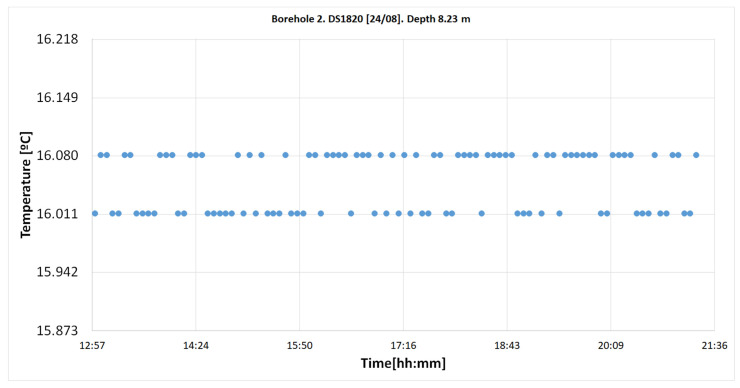
Temperature records on 23 August in the “Q-Thermie-Uniovi.2” borehole with DS18B20 sensor at 8.23 m. It can observed that 53.4% of the measurements are at 16.011 °C and the remaining 46.6% are at 16.080 °C, with a temperature difference of 0.069 °C.

Figure 30 represents the temperature values at 8.23 m, between 12:55 am and 9:21 pm hours. The number of measurements was 103.

##### Temperatures from Sensor S10.31

**Figure 31:**
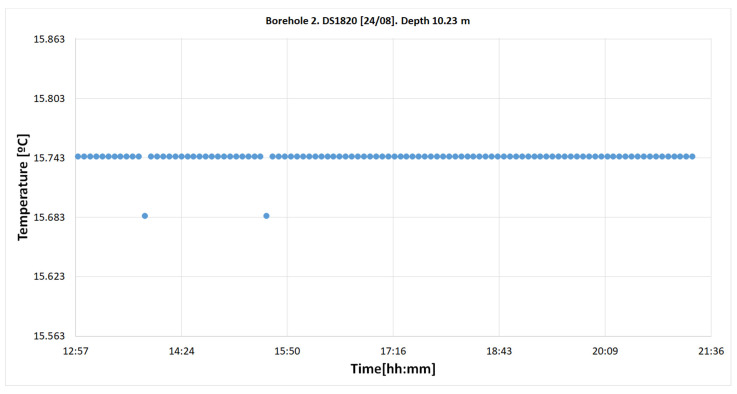
Temperature register on 23 August in the “Q-Thermie-Uniovi-2” borehole with DS18B20 sensor at 10.31 m, obtained via interpolation.

Figure 31 presents the temperature values at 10.31 m between 12:55 am and 9:21 pm. The number of measurements was 103, e.g., for S8.23.

The frequency diagram shows that 98.06% of the measurements were at 15.744 and that the remaining 1.94% were at 15.684 °C, with a thermal jump of 0.060 °C.

It is usual for the temperature to be more stable at deeper points (10.23 m) than at a shallower depth.

##### Calculated Temperatures at 9.5 m

**Figure 32:**
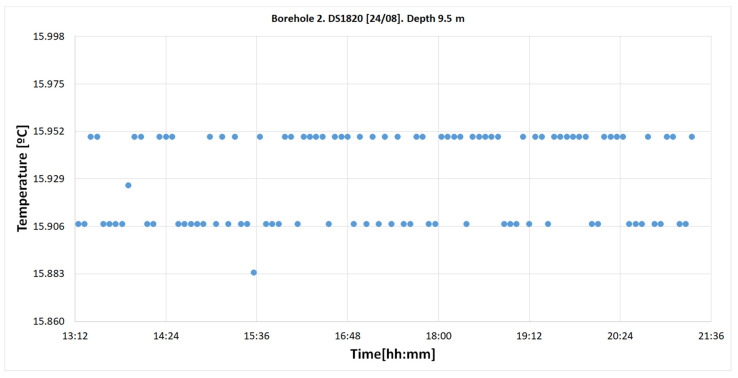
Temperature registers on 23 August in the “Q-Thermie-Uniovi-2” borehole with DS18B20 sensor at 9.5 m, obtained via interpolation.

In order to compare the results from the two sensor types, placed in different locations and depths, the temperature of a fictitious DS18B20 sensor located at 9.5 m was calculated. This was achieved by interpolating the measurements between 8.23 and 10.31 m. The results from this interpolation are represented in Figure 32.

#### 4.2.3. Comparative Results

In summary, temperature values obtained from both type of sensors in borehole two are shown (Table 6). The temperature differences were small, even if they were not in the same position in the borehole.

### 4.3. Measurements Registered by the Sensors

**Figure 33:**
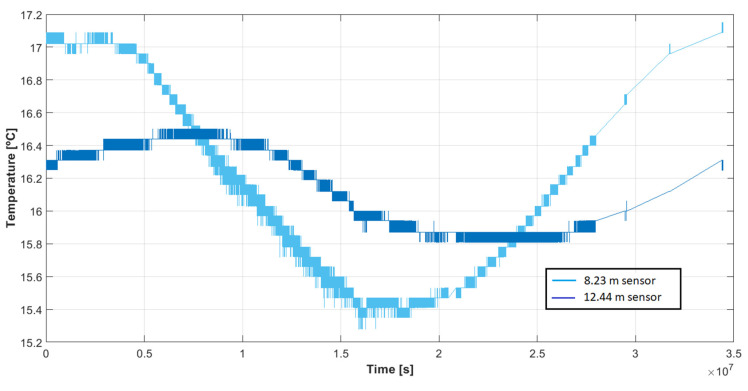
Temporal evolution of temperature at depths 8.23 and 12.44 m for the sensor DS18B20 over 405 days.

As shown by Figure 33, the temporal evolution of temperature at depths 8.23 and 12.44 m for the sensor DS18B20 spanned over 405 days. This figure shows that the measurements were reliable and coherent, and that they could be applied to determine thermal properties of the ground, such as thermal conductivity or thermal diffusivity, as demonstrated in [32]. The amplitude attenuation of the temperature with depth could be observed—e.g., in 4.21 m, and the variation reduced by 0.6 °C. Moreover, the lag of the temperature wave with depth could also be observed, as forecasted in a theoretical study [30].

The presented instrumentation could be applied to measure temperatures at different depths during a TRT test. Moreover, it could be used to monitor the evolution of a shallow geothermal installation over long periods of time.

## 5. Discussion and Conclusions

Two types of simple and inexpensive devices to measure and register temperature over long periods of time, under geothermal borehole conditions, are presented.

All the components are standard, though the set-up in the geothermal borehole is a novel application and can provide useful information in this type of studies.

The results from the study can be summarized as follows:

The instrumentation is built with standard components to register ground temperature. This set-up is simple, reliable, and robust, and it has sufficient precision to provide basic information in shallow geothermal studies.

The calibration procedure for the sensors in the 1-wire system is detailed.

- Thermal jumps:

In the “Q-Thermie-uniovi-1” borehole, at a depth of 9.5 m, the temperature is registered with the following differences:

  ○ The TMCx-HD sensors produce thermal jumps of 0.024 °C;
  ○ The DS18B20 sensors produce thermal jumps of 0.0625 °C.

In the “Q-Thermie-uniovi-1” borehole 2, the temperature is registered with the following differences:

  ○ The thermal jump for the TMCx-HD at 9.5 m is 0.024 °C;
  ○ The thermal jump for the DS18B20 sensors located at 8.23 and 10.31 m is 0.069 °C and 0.0601 °C, respectively.

- Sensor position:
  ○ In the “Q-Thermie-uniovi-1” borehole, both types of sensors are at the same depth and the same relative position, i.e., on the outside of the outer geothermal pipe.
  ○ The TMCx-HD sensors register temperatures of 16.344 °C, while the DS18B20 sensors register temperatures of 16.062 °C, showing a difference 0.282 °C. The 1-wire is not calibrated; hence, the temperatures which should be the same (since they are measured in the same position) are not.
  ○ In the “Q-Thermie-uniovi-2” borehole, the sensors are in different relative positions. Those of the TMCx-HD type are located on the outer face of the geothermal pipe, embedded in the geothermal fill, while the 1-wire DS18B20 sensors are on the geometric axis of the 50 mm geothermal tube.

The TMC sensor is located at 9.5 m and the DS18B20 sensors closest to 9.5 m are located at 8.23 m and 10.31 m, respectively.

The temperature measured by the TMC at 9.5 m is 15.939 °C.

The 1-wire is calibrated, and the measured temperatures are corrected. The temperature interpolated for the fictitious DS18B20 sensor at 9.5 m ranges between 15.906 and 15.952 °C, thus agreeing with the temperature given by the TMC sensor.

Nevertheless, the applied system fulfils all the requirements regarding precision and reliability, as it can detect temperature differences smaller than 0.1 °C. In addition, it has a small size, and it can set up the space between the pipe and borehole walls. Finally, DS18B20 sensors continue to register temperature after several years.

## Figures and Tables

**Table 1:** TMCx-HD sensor locations for the two boreholes.

Borehole Depth (m)	
Q-Thermie-Uniovi-1	Q-Thermie-Uniovi-2
1.5	1.5
4.5	4.5
9.5	9.5

**Table 2:** DS18B20 sensor depths and distance between sensors in both boreholes.

Q-Thermie-Uniovi-1		Q-Thermie-Uniovi-2	
Depth at the Borehole (m)	Distance between Sensors (m)	Depth at the Borehole (m)	Distance between Sensors (m)
On the surface		On the surface	
1.60		1.67	
4.00	2.36	3.86	2.19
		6.08	2.22
		8.23	2.15
9.40	5.40	10.31	2.08
		12.44	2.13
15.02	5.62	14.55	2.11
		16.72	2.17
		18.01	1.29
20.04	5.02	20.14	2.13
		22.17	2.03
25.02	4.98	24.22	2.05
		26.26	2.04
		28.38	2.12
30.70	5.68	30.44	2.06
		32.50	2.06
35.80	5.10	34.61	2.10
		36.75	2.15
		38.85	2.10
41.40	5.60	40.98	2.13
		43.41	2.43
46.50	5.10		

**Table 3:** Mean temperatures of each zone.

Zone Number	Mean Temperature $\left(T_{m}\right)$	Assessment of Zone Temperature
Zone 1	16.24 °C	Stable
Zone 2	14.98 °C	Stable
Zone 3	15.85 °C	Variable
Zone 4	16.69 °C	Stable

**Table 4:** DS18B20 calibration results.

	Sensor 1.67 m	Sensor 3.86 m	Sensor 6.08 m	Sensor 8.23 m	Sensor 10.31 m	Sensor 12.44 m	Sensor 14.55 m
*a*	0.995	1.0007	1.0074	0.989	1.0074	1.004	0.999
*b*	0.042	0.1458	−0.0979	0.127	−0.3203	−0.008	−0.054
*R* ^2^	0.998	0.9985	0.9985	0.998	0.998	0.998	0.998
	Sensor 16.72 m	Sensor 18.01 m	Sensor 20.14 m	Sensor 22.17 m	Sensor 24.22 m	Sensor 26.26 m	Sensor 28.38 m
*a*	0.999	0.993	0.991	0.9996	0.992	0.994	1.004
*b*	0.1748	0.076	0.048	0.102	0.144	0.358	−0.285
*R* ^2^	0.995	0.994	0.997	0.998	0.996	0.998	0.997
	Sensor 30.44 m	Sensor 32.50 m	Sensor 34.61 m	Sensor 36.75 m	Sensor 38.85 m	Sensor 40.98 m	Sensor 43.41 m
*a*	0.9996	0.9984	0.998	0.9865	0.9897	0.997	1.017
*b*	−0.05	0.0023	0.1339	0.2833	0.07	0.1167	−0.3402
*R* ^2^	0.998	0.998	0.9976	0.9979	0.997	0.998	0.9965

**Table 5:** Borehole 1. Temperature comparison between the two sensor types.

Sensor Type	Depth (m)	Temperature Measured (°C)	Difference (°C)
TMC	9.5	16.344–16.368	0.282
DS1820	8.23	16.062	
Potential cause of the temperature difference	Lack of calibration of DS18B20 sensors		

**Table 6:** Temperature comparison between the two sensor types.

Sensor Type	Depth (m)	Temperature Measured (°C)	Temperature Estimated (°C)
TMC	9.5	15.939	
DS1820	8.23	16.089–16.011	
	10.31	15.744	
			15.906–15.952
Temperature differences = 0.013–0.033 °C			

## Data Availability

Not applicable.
